# Use of an audience response system to maximise response rates and expedite a modified Delphi process for consensus on occupational health

**DOI:** 10.1186/s12995-016-0098-5

**Published:** 2016-03-02

**Authors:** Tar-Ching Aw, Tom Loney, Anza Elias, Soha Ali, Balázs Ádám

**Affiliations:** Institute of Public Health, College of Medicine and Health Sciences, United Arab Emirates University, PO Box 17666 Al Ain, United Arab Emirates; PAPRSB Institute of Health Sciences, Universiti Brunei Darussalam, Japan Tungku Link, Gadong, BE1410 Brunei Darussalam; Department of Preventive Medicine, Faculty of Public Health, University of Debrecen, PO Box 9, Debrecen, H-4012 Hungary

**Keywords:** Audience response system, Consensus, Delphi technique, Occupational health

## Abstract

**Background:**

Different methods have attempted to obtain consensus on occupational health issues. The objective of this paper is to describe a modified three-stage Delphi process that uses a wireless audience response system to enable consensus to be attained in a single day and to maximise response rates. The modified Delphi approach required: a) agreeing the topic/s of interest for which consensus is sought, b) identifying key stakeholders whose opinions are required; c) assembling the stakeholders for a one-day event. Participants’ opinions were recorded primarily through use of a system of individual wireless audience response devices (‘clickers’) linked to a computer. Providing immediate feedback enabled the audience to consider the group’s views before proceeding to the next stage. From an initial round of responses, participants were asked to narrow their choices to any five preferred options. A third round was conducted, using the ‘clickers’ to rank 5 of the most popular group options. Through this iterative exercise, stakeholder consensus was achieved after three decision rounds.

**Results:**

The use of the modifications and the wireless audience response system described enabled stakeholders to provide a group view on specific occupational health issues e.g. priorities or barriers or resources needed. Completing the three-stage iterative exercise in a day maximised the response rate with advantages for both the participants and the researchers. Careful design of the protocol is essential, with a team familiar with information technology to ensure smooth execution of the various stages.

**Conclusions:**

Modification of the Delphi method with the use of a wireless audience participation system facilitates rapid consensus.

## Background

The Delphi method was originally developed by the US Rand Corporation as a method for decision making and forecasting [1–3]. The technique used an iterative approach for obtaining consensus. It appeared to be especially useful in the absence or non-availability of definitive data, and when expert opinion is therefore sought as a possible alternative to decisions based on published evidence. The subject area in which it was first used was defence, but the technique was then adopted by other disciplines, such as business, social science and medicine for research, education and training purposes [4–10]. The Delphi approach allowed agreement to be reached, even in areas as potentially contentious as determination of priorities, provision of services and barriers to delivery. The technique has also been used for occupational health in several countries, including Malaysia, the United Arab Emirates and the United Kingdom [11–15].

One of the limitations of the multi-stage iterative process is the low response rate, especially during the subsequent rounds of iteration, as it has been experienced in various settings [16, 17]. This has led to considerations as to whether there is a possibility of completing the exercise in a shorter period of time to reduce poor participation from the first round through to completion. Single-round, almost real-time methods and use of online systems have been proposed [18, 19], and researchers have attempted to modify the method to improve participation rates and shorten the process time. We have trialled the use of a computerised audience-response system for determining key issues and barriers to effective provision of occupational health in the United Arab Emirates (UAE) [20]. The method was refined and used in 2014 to determine consensus on health and safety issues and obstacles to successful implementation of health and safety programmes for a large utilities company in Abu Dhabi.

## Methods

The Delphi approach was modified to obtain results in a short time, requiring only one day of direct contact with stakeholders. This is possible with the use of a back-up computer team, and through the use of an automated audience response system to obtain almost instantaneous feedback. The modified process involves several stages:

- Identification of the topic of interest
  Two occupational health projects using the modified Delphi method were conducted in Abu Dhabi (in 2009 and 2014). The two organisations involved were a government health authority (2009), and a large utilities company (2014). In 2009, the purpose was to obtain a consensus view on three key areas of occupational health and safety in the United Arab Emirates. These were i) specific priority issues, ii) data gaps and iii) resources needed. In 2014, the aim was to determine within the company i) priorities, and ii) barriers to success, in developing and implementing provisions for occupational health and safety.
- Selection of participants
  Stakeholders were identified by the organisations involved to participate in the Delphi exercise. The main requirement was that they should represent a spectrum of those involved or with a major interest in occupational health and safety. The 2009 project included 64 stakeholders with a balanced representation from employers and employees, government officials, representatives of industry, academics and other health and safety practitioners in the United Arab Emirates. In the 2014 exercise the participants consisted of 80 managers, safety representatives and members of the workforce at different seniority levels and from different geographical regions served by the organisation (i.e. Eastern, Western and Central region of the Emirate of Abu Dhabi).
- Listing of options
  The initial step in our modified Delphi method involved the selected participants listing as many options as possible in regards to the topic of interest. In 2009, this was completed on a single blank sheet of paper that was then collected for immediate analysis and tabulation of all the responses. Thirty-two specific priority issues and ten items each as data gaps and resources required were listed. In 2014, a list of 20 priorities and 20 barriers for occupational health was available for the organisation from a recent questionnaire survey of approximately 750 employees. The items were entered into a computer program linked to the automated audience response system.
- Use of the audience response system
  The 2009 study used a three-stage iterative process. Some adjustments to the procedure were made in 2014, based on the experience and feedback from the 2009 exercise. Due to substantial overlap, only the methodological details of the improved three-stage process used in the 2014 study are discussed below.

**Fig. 1:**
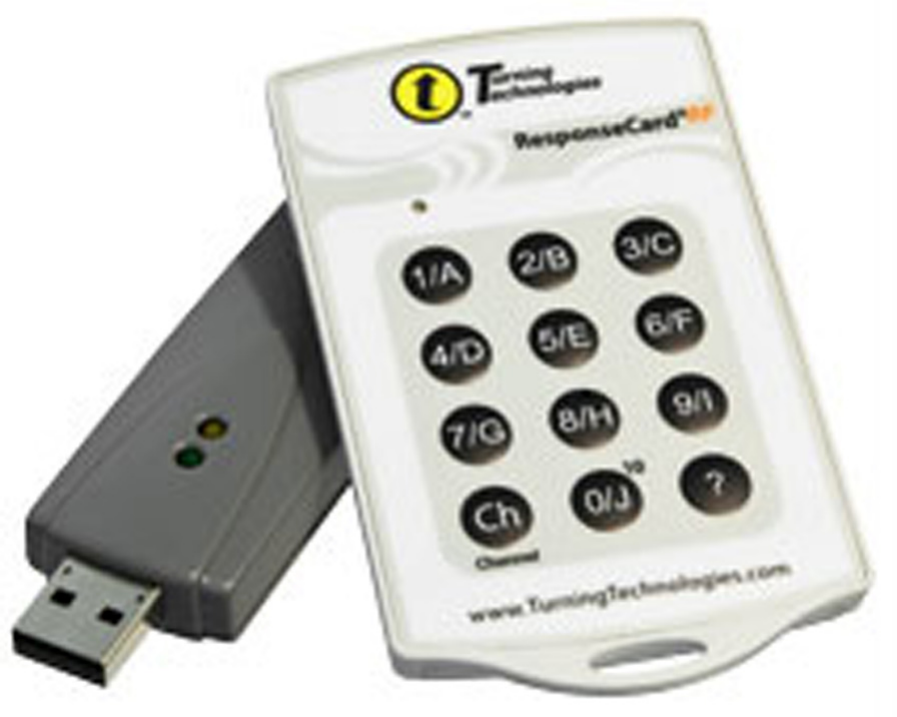
Photo of a ‘clicker’ as part of the audience response system hardware

On the day of the exercise, the stakeholders were instructed on the necessity to provide their own views without involving any discussion with their colleagues. The importance of obtaining a personal individual opinion was stressed. Each participant was provided with a ‘clicker’ (Keepad Interactive, NSW, Australia; see illustration, Fig. 1) and received oral instructions and practice sessions on how to use the device.

The personal wireless ‘clicker’ allowed individuals to make a choice from several options, with their choices logged directly onto a computer.

- Round 1: Indicating the relevance of the pre-identified items
  The participants were asked to indicate on a pre-printed color-coded single-sheet form whether they felt that each of the 20 items (separately for priorities, and then for barriers in the case of the 2014 project) were very relevant, somewhat relevant or of minor relevance. The participants were then required to use the information that they had provided on the forms to indicate their choice by use of the ‘clicker’, so that the group’s views are summarised automatically through the computer, thereby providing quick analysis for the research team and feedback to the audience on the views of the group at the appropriate stage of the modified Delphi process.The data from stage 1 were analysed to exclude approximately one third (33 %) of the listed priorities and barriers from further consideration. About one third of the items, those with the highest number of participants indicating that they were of ‘minor relevance’, were discarded.
- Round 2: Choosing the five most preferred items
  Following exclusion of a third of the initial options, a shorter list of items was printed on a single sheet of color-coded paper and distributed to the audience. Next, the participants from the utilities company were instructed to select only five of their most preferred choices from each list (13 residual priorities items and 12 barriers). The selections were again transferred to the computer through use of the audience response system and the five items indicated by the highest number of participants as their preferred choice were determined. The selections of the audience were displayed on screen through the computer to provide feedback on the views of the group before the next stage.
- Round 3: Ranking the five items most favoured by the group
  The five most preferred items by the whole group for both priorities and barriers were distributed on color-coded paper and participants were asked to rank these five items. The audience response system was then used to determine the rank of each item on a scale from 1 to 5 with 1 indicating the most important. The final rank of the top five occupational health issues for priorities and the top five barriers was determined by the average rank of the items voted by the participants.

The study was approved by the Social Sciences Research Ethics Committee of the United Arab Emirates University.

## Results

### Study participants

In 2009, the 64 participants consisted of 23 (38 %) individuals from government agencies, 17 (28 %) from industry, and 20 (33 %) from other groups (including academia, health and safety practitioners and employee interest groups). In 2014, the 80 participants were all employees of the utilities company. Fifty-five (69 %) were mainly office-based staff and the remaining 25 (31 %) were either only field-based or largely field-based with some office-based duties. They represented different levels of seniority (the majority, 50 %, working in middle management) and worked in several different geographical regions (i.e. Eastern, Western and Central region of the Emirate of Abu Dhabi). Thirty-eight (48 %) of the participants worked in the power supply division of the company, whilst fourteen (18 %) were in the water supply division, and twenty-seven (34 %) in customer services and other smaller sections.

### Response rate

**Fig. 2:**
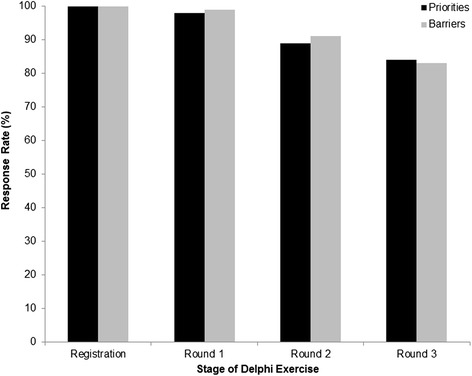
Response rate (%) for three-round Delphi study conducted in 2014

In 2009, the participation rate was 94 % in our three-stage Delphi project. There were slight changes in the number of respondents during the three stages of the 2014 Delphi exercise. Altogether 80 employees participated in the study with 14 leaving during the process and a two arriving after it had been started. There were 78 (98 % of the total) and 79 (99 %) responses in the first stage of indicating relevance of priorities and barriers, respectively (Fig. 2). A small drop in the response rate was experienced in the second stage to 89 and 91 %, respectively (Fig. 2). The decrease continued in the last stage; 84 % completed ranking of the top five priorities and 83 % for the top five barriers (Fig. 2).

### Final ranks

The 2009 Delphi exercise identified the top five priority areas, data gaps and resource needs for the UAE in occupational health and safety (Table 1).

**Table 1:** Top five priorities, data gaps and resource needs for the United Arab Emirates in occupational health and safety, Delphi exercise, 2009

Priorities	Data gaps	Resource needs
1. Adequate occupational health and safety legislation	1. Absence of workplace injury reporting system	1. Government support and commitment for occupational health and safety
2. Availability of guidelines on health and safety	2. Lack of accurate health and safety statistics	2. National guidelines on industry-specific health and safety standards
3. A central government authority for developing occupational health strategy	3. Data on the extent and results of occupational health screening	3. Education and training for different groups of health and safety practitioners
4. Competency of occupational health professionals	4. Data on occupational diseases	4. A central health and safety resource
5. Prevention of work-related illness and injury	5. Information on occupational exposure assessment	5. A system for collecting occupational health and safety statistics

The top five priorities and barriers of occupational health and safety determined by the 2014 Delphi study for the utilities company are shown in Table 2.

**Table 2:** Top five priorities and barriers of occupational health and safety for a utilities company in the United Arab Emirates, Delphi exercise, 2014

Priorities	Barriers
1. Personal protective equipment	1. Lack of management commitment
2. Training	2. Production taking priority over safety considerations
3. Quality of work equipment	3. Workload
4. Regular safety inspections	4. Poor communication regarding health and safety
5. First aid facilities and training	5. A ‘blame’ culture

There was an expected difference in consensus regarding priorities for health and safety for the country, versus priorities and barriers for health and safety within a company.

## Discussion

The two exercises in Abu Dhabi showed that the modified Delphi method using an automated audience response system had many advantages in obtaining a consensus view from stakeholders on pre-determined issues in occupational health, most importantly the exercise could be completed in a short period of time with a very high response rate in the consecutive rounds. The use of a combination of written responses and the computerised audience response system proved effective in enabling a multi-round iterative exercise to be conducted over a day, thereby reducing dropouts from participation. The majority of previous Delphi projects have taken several weeks or months [5, 14]. This was because several iterations were required, and a postal or online system was used to gather information, with time allocated between iterations in order for responses to be received.

The computer-aided audience response system also enabled almost immediate feedback to the group about their responses at specific stages of the modified Delphi study. Stages 2 and 3 of the iterative process could then proceed after participants had received feedback from the preceding stages, allowing them for comments and questions. At the end, the final results of the exercise were shared with the participants so that everybody could express their final opinion. Informal feedback through initial provision of the findings to the company suggests that there was general agreement with the priorities and barriers identified through the Delphi exercise.

Completion of all stages in a day reduced the dropout rate. The final participation rate for multi-stage Delphi exercise is often low and would be affected by the number of rounds involved, and the time given between stages. A recent study employed a two-round online Delphi study to develop consensus on remote healthcare practitioners’ competency for oil and gas operations and the valid response rate for round one and two was 27 and 24 %, respectively [17]. Study authors reported that technical issues with the recruitment, sampling and data collection techniques accounted for 59 % of the non-response across the two rounds: out-of-office replies (26 %), incorrect email addresses (19 %), and burst rate limit exceeded (14 %) [17]. In the two-stage postal exercise on priorities for occupational health research involving 53 senior occupational physicians, Harrington obtained an 86 % response to round 1, and 91 % for round 2 [12]. When the exercise was repeated as a postal questionnaire to personnel managers, the response rate was 24 % despite two reminders and a second mailing to non-responders. For our three-stage Delphi project in 2009, the participation rate was 94 %, and in 2014, 83 % of registered participants completed all three stages of the exercise. With a postal questionnaire the participation rate often decreases after each round. Follow up of non-responders is difficult if there are concerns about preservation of the confidentiality of responses. With our modified system, all participants were requested to remain until all stages were completed. Despite the whole process taking only one day, a 17 % drop-out rate was still experienced, with a few individuals leaving before completing either stage three or stages two and three. This may be partly due to inadequate communication or commitment by the participants regarding the importance to remain till the end of the exercise.

Although neither the Delphi process nor the computerised audience response system was familiar for the participants, the majority could understand and complete the expected tasks. The modified approach enabled individual views to be expressed, with equal weighting given to each person’s opinion, regardless of position or rank within the organisation, or professional or managerial background or experience. The likelihood of opinions being influenced by the views of senior or more vocal colleagues was reduced by providing an individual ‘clicker’ for each participant. Individual participant choices could then be transmitted to the computer with preservation of anonymity. Participants were reminded to provide their own opinions without group discussion, and that the projects were designed to obtain individual opinion and not necessarily company policy or views of the group. Therefore, there was a strong likelihood that the views of individuals were obtained. With postal or even online questionnaires there is no certainty as to whether the views expressed or the choices made were completed as a group exercise or completed by persons other than the identified respondent.

A major limitation of the modified approach is the requirement for stakeholders to be physically present to participate for a day. This requires time away from work, and the costs of accommodating a large number of individuals including finding a suitable venue and providing meals and refreshments. This was feasible in the two projects in Abu Dhabi as there was commitment and support from the employers for the process. There is also a need for a small team of experienced support staff to facilitate the distribution and collection of questionnaires and ‘clickers’, and to analyse data captured by the audience response system in a limited time between the stages. The availability of a set of 100 clickers (to include spare devices) that had to be checked and tested ahead of the exercise was essential. The process required a smooth transition from one stage of the Delphi procedure to the next, without unnecessary delay that could well reduce the likelihood of participants remaining for subsequent stages. Good audio-visual facilities were essential, and a preliminary explanation as to the purpose of the process, and the importance of full participation was key to the success of the modified Delphi study.

The selection of participants was not random therefore it did not assure that opinions were representative for the views of the organisations they represented. Nevertheless, random selection is not the main purpose of selecting participants for a Delphi exercise; rather, purposeful sampling was used to recruit knowledgeable and experienced participants on the topic of interest [10]. This concept was obtained by inviting acknowledged professionals from the field of occupational health and safety available in the United Arab Emirates in the 2009 study. In 2014, the organisers of the initial survey within the company were entrusted to recruit employees related to occupational health and safety issues from various sectors of the company in a number that is manageable in a one-day exercise. The choice of participants with different levels of seniority, representing different professional groups or working in different geographical areas allowed views to be gathered from a wide area of representation.

## Conclusions

The presented modified Delphi technique with the use of a wireless audience participation system enables rapid consensus that proves especially advantageous in effectively identifying priorities and barriers of occupational health and safety in countries and workplace settings where such knowledge is particularly needed.

## References

[1] Dalkey N, Helmer O (1963). An experimental application of the Delphi method to the use of experts. Manag Sci.

[2] Gupta UG, Clarke RE (1996). Theory and applications of the Delphi technique: a bibliography (1975–1994). Technol Forecast Soc Chang.

[3] Linstone HA, Turoff M (2011). Delphi: A brief look forward and backward. Technol Forecast Soc Chang.

[4] MacDonald E, Ritchie K, Murray K, Gilmour W (2000). Requirements for occupational medicine training in Europe: a Delphi study. Occup Environ Med.

[5] van der Beek AJ, Frings-Dresen MH, van Djik FJ, Houtman IL (1997). Priorities in occupational health research: a Delphi study in the Netherlands. Occup Environ Med.

[6] Van Djik JAGM (1990). Delphi method as a learning instrument. Technol Forecast Soc Chang.

[7] Landeta J (2006). Current validity of the Delphi method in social sciences. Technol Forecast Soc Chang.

[8] Powell C (2002). The Delphi technique: myths and realities. J Adv Nurs.

[9] Graham B, Regehr G, Wright JG (2003). Delphi as a method to establish consensus for diagnostic criteria. J Clin Epi.

[10] Okoli C, Pawlowski SD (2004). The Delphi method as a research tool: an example, design considerations and applications. Inform Manag.

[11] Agius RM (1993). Priorities for managing occupational allergy – a Delphi consensus. Clin Exp Allergy.

[12] Harrington JM (1994). Research priorities in occupational medicine: a survey of United Kingdom medical opinion by the Delphi technique. Occup Environ Med.

[13] Harrington JM, Calvert IA (1996). Research priorities in occupational medicine: a survey of United Kingdom personnel managers. Occup Environ Med.

[14] Sadhra S, Beach JR, Aw TC, Sheikh-Ahmed K (2001). Occupational health priorities in Malaysia: a Delphi study. Occup Environ Med.

[15] Loney T, Aw TC. Development of occupational health in the Gulf Cooperation council countries: The UAE experience. J Inst Remote Hlth Care. 2014;5(1):18–24.

[16] Reetoo KN, Harrington JM, Macdonald EB (2005). Required competencies of occupational physicians: a Delphi survey of UK customers. Occup Environ Med.

[17] Klein S, Mohamed H (2014). Developing consensus on remote healthcare practitioners’ competency for oil and gas operations: A Delphi study: Institute of Remote Healthcare: conference proceedings, October 07–08, 2014.

[18] Gordon T, Pease A (2006). RT Delphi: An efficient, “round-less” almost real time Delphi method. Technol Forecast Soc Chang.

[19] Steinert M (2009). A dissensus based online Delphi approach: An explorative research tool. Technol Forecast Soc Chang.

[20] Aw TC (2010). Occupational health and safety priorities for the UAE. Internal report for the Health Authority of Abu Dhabi.

